# What we (should) talk about when we talk about fruitfulness

**DOI:** 10.1007/s13194-018-0231-7

**Published:** 2018-10-26

**Authors:** Silvia Ivani

**Affiliations:** Tilburg University, Dante Building, Postbus 90153, 5000 LE Tilburg, The Netherlands

**Keywords:** Fruitfulness, Cognitive values, Evolutionary psychology, Methodological Adaptationism

## Abstract

What are the relevant values to the appraisal of research programs? This question remains hotly debated, as philosophers have recently proposed many lists of values potentially relevant to scientific appraisal. Surprisingly, despite being mentioned in many lists, little attention has been paid to fruitfulness. It is unclear how fruitfulness should be explicated, and whether it has any substantial role in scientific appraisal. In this paper, I argue we should explicate fruitfulness as the capacity to develop of research programs. Moreover, I provide a novel strategy to assess and compare the fruitfulness of programs focused on their research questions and heuristics. To illustrate how this strategy would work, I will discuss a case study, namely the adaptationist program in evolutionary psychology.

## Introduction

Thomas Kuhn (1977) suggested a list of five values that, among others, have an actual, beneficial role in scientific appraisal. This list includes accuracy, consistency, scope, simplicity, and fruitfulness. Philosophers have then proposed several lists that include these classical values (e.g. McMullin 1982; Douglas 2009). Nonetheless, there is not a general consensus about the definitions and roles of these values. While many papers have shed light on some of these values, such as simplicity (e.g. Forster and Sober 1994; Baker 2003), little attention has been paid to other values, such as fruitfulness. Few studies have addressed the explication of fruitfulness as a value relevant to assess and compare rival research programs. Much of the literature provides definitions that are too vague for providing strategies to assess the fruitfulness of programs. A good illustration of this problem is Kuhn’s account of fruitfulness. Despite ascribing a prominent role to fruitfulness in theory choice, he spent very few words on it. When defining it, he just states that fruitful theories “disclose new phenomena or previously unnoted relationships among those already known” (1977, 322). Moreover, there is considerable disagreement over its importance in theory assessment. While some philosophers argue that fruitfulness is a crucial value for the assessment of programs (McMullin 1976; Kuhn 1977), others claim that it plays a secondary role in theory appraisal (Nolan 1999; Douglas 2009). For instance, Hugh Lacey (1999) argues that fruitfulness should not be included in the list of values that are relevant for assessing theories and programs. According to Lacey, fruitfulness “pertains more to such stances as the provisional entrainment of theories rather than to their acceptance” (1999, 60). Fruitfulness has also been criticized as having a noxious influence on science. Helen Longino (1995) criticizes the Kuhnian notion of fruitfulness because it focuses on the development of the content of theories in response to puzzles that have to be solved. Instead, the development of a program should be assessed as valuable, Longino argues, if it “can be used to improve living conditions in a way that reduces inequalities of power” (1995, 394).^1^

Overall, despite being frequently mentioned by philosophers and scientists, how to understand fruitfulness remains obscure. A satisfactory explication should help articulate concrete strategies for assessing and comparing the fruitfulness of research programs. It should then answer at least two questions: What is fruitfulness? What are reliable strategies for assessing and comparing the fruitfulness of rival research programs in science? The aims of this paper are to provide an explication of fruitfulness and a strategy to assess it.

Despite being vague, the literature on fruitfulness provides useful insights for developing an in-depth study. Indeed, many of the accounts suggested so far share the core idea that fruitfulness would concern the desirable development involving changes, extensions, and improvements of research programs’ content. In this view, fruitful programs can produce epistemic goods. I argue that in order to provide a satisfactory explication of fruitfulness, this core idea should be maintained and developed further. In particular, I suggest assessing fruitfulness by taking into account the set of tools that makes the development possible. This set is what scientists can employ for developing the content of the program. These are the tools for formulating and testing hypotheses, such as experiments and methods to analyse data. I argue that focusing on two of these tools, namely the research questions and discovery heuristics, provides us with a concrete, simple strategy for evaluating and comparing the fruitfulness of research programs. Considering research questions and discovery heuristics constitutes a novel approach to fruitfulness. To date, the literature has failed to recognize these factors as key elements for the assessment of fruitfulness.

This paper is structured as follows. In the second section, I consider the literature on fruitfulness and I illustrate the aforementioned core idea as presented by Thomas Kuhn, Ernan McMullin, Daniel Nolan, and Samuel Schindler. Furthermore, I outline my approach to assess fruitfulness, which is focused on research questions and discovery heuristics. In the third section, I introduce a case study, namely the adaptationist program in evolutionary psychology. This case study shows how considering questions and heuristics can clarify and simplify the assessment of fruitfulness of research programs. In the fourth section, I draw conclusions.

## What is fruitfulness?

Scientists frequently refer to fruitfulness as an important feature of research programs.^2^ Max Planck talks about the fruitfulness of explaining entropy by referring to the probability calculus in physics (1915, 35). Henri Poincarè praises the fruitfulness of mathematical reasoning (1905, xix). The evolutionary psychologist David Buss stresses how research on sex differences has become more fruitful by focusing on (internal) psychological mechanisms rather than on (external) manifest behaviours (1998, 22). In particular, Buss claims that this shift was fruitful because it led to the formulation of several hypotheses and the discovery of many sexual differences. Stephen Gould, instead, criticizes evolutionary psychology as fruitless. He claims: “Evolutionary psychology could, in my view, become a fruitful science by replacing its current penchant for narrow, and often barren, speculation with respect for the pluralistic range of available alternatives” (1997). There are many similar examples that could be cited. However, the pervasiveness of fruitfulness in scientific papers is not mirrored in the philosophical literature. Relatively little attention has been paid to explicating fruitfulness and its role in theory appraisal. One of the consequences of the lack of a clear explication is that fruitfulness can be virtually ascribed to many different programs, even rival ones. The fact that we can ascribe fruitfulness to rival programs does not constitute a problem. Indeed, two rival programs can be both fruitful, just as they can be both simple or internally consistent. However, the problem with fruitfulness is that it can be *easily* ascribed to many programs because its definition is loose and no clear strategy for assessing it is provided.^3^ As the above examples of Buss and Gould show, scientists may radically disagree whether a program is fruitful or not. This may depend on the divergent ideas that scientists entertain about the merits of a research program. However, in the next sections I argue that such disputes may be fostered by a vague definition of fruitfulness and that it is possible to shed light on them by appealing to a clear account of fruitfulness.

In the following pages, I explicate a notion of fruitfulness understood as a capacity to develop of programs. However, before doing that, in the next subsection I discuss the accounts suggested by Thomas Kuhn, Ernan McMullin, Daniel Nolan, and Samuel Schindler. While Kuhn and McMullin provide two of the earliest attempts to explicate fruitfulness, the accounts of Nolan and Schindler are two of the rare recent analyses of fruitfulness.

### Fruitfulness as desirable development of programs

Thomas Kuhn includes fruitfulness in his pioneering list of values (1977). Although he claims that fruitfulness is “of special importance to actual scientific decisions” (1977, 322), he spent very few words on this value. Nonetheless, from the little he says, it is clear that he construes fruitful programs as producing new research findings and disclosing “new phenomena or previously unnoted relationships among those already known” (ibid.). In particular, fruitful programs produce new findings and discoveries that help to reach solutions to research puzzles. For instance, Kuhn claims that the adoption of mechanico-corpuscolar explanations in different programs made them fruitful since it led to the solutions of “problems that had defied generally accepted solution and suggesting others to replace them” (1962, 104).

Fruitfulness plays a crucial role in the Kuhnian picture of theory choice, influencing scientists’ decision leading to transitions from old paradigms to new paradigms.^4^ Paradigms are, in Kuhn’s view, puzzle-solving exemplars, determining the rules for solving puzzles and what counts as an acceptable solution (1962, 38). When a paradigm and its rules persistently fail to solve puzzles, other paradigms and new rules emerge and scientists become interested in these other programs. Hence, the power of solving puzzles, i.e. the fruitfulness of programs, is one of the main reasons that convince scientists to work with a paradigm.

Kuhn’s ideas have influenced other philosophers’ understanding of fruitfulness. For instance, Ernan McMullin elaborates the idea of fruitfulness as the power of programs to develop and solve puzzles and anomalies. Indeed, in his view “a fertile theory is one with the capacity to grow and change” (1979, 60) by finding solutions to puzzle and anomalies.^5^ Such a capacity is a diachronic virtue of programs, i.e., it manifests itself over the course of time (McMullin 2009, 505). To account for this time-dependent aspect, McMullin proposes two kinds of fruitfulness, namely proven fertility and untested fertility. While proven fertility is past oriented and it concerns the past success of a program in growing and changing, untested fertility is future oriented and refers to the potential ability to develop in the future (McMullin 1976, 400). McMullin does not ascribe equal importance to the two kinds of fruitfulness. Indeed, he argues that while proven fertility is a truth-indicative feature of programs, untested fertility is not and, because of this, focuses his analysis on proven fertility.^6^

McMullin explicates fruitfulness as follows:

“The [fruitful] theory proves able to make novel predictions that were not part of the set of original explananda. More important, the theory proves to have the imaginative resources, functioning here rather as a metaphor might in literature, to enable anomalies to be overcome and new and powerful extensions to be made. Here it is the long-term proven ability of the theory or research program to generate fruitful additions and modifications that has to be taken into account.” (McMullin 1982, 16)

In this view, fruitful programs have the adequate instruments to promote the flowering of new ideas and extensions. They stimulate the creative imagination of scientists to modify the content of programs in desirable ways (McMullin 1979, 60). As Segall explains, according to McMullin fruitful programs possess metaphorical suggestiveness: just like good metaphors, fruitful programs suggest creative ideas to improve our understanding or knowledge of the world (Segall 2009, 58). However, being fruitful does not mean encouraging mere change. A type of change, McMullin argues, is of special importance. He suggests focusing on the modifications of a program occurring when it has to deal with anomalies, which he calls non-*ad hoc* accommodations. These modifications are valuable since they involve adjustments that do not modify the fundamental ideas of the program (McMullin 1985, 264). By way of illustration, McMullin discusses the discovery of the electron spin in the Bohr model. Scientists were not able to explain some anomalies, i.e., the reaction of hydrogen to a strong magnetic field. Then, such anomalies were successfully accommodated by postulating electron spin. McMullin claims:

“Electron spin was not part of the original Bohr model. Its omission was not a formal idealization, strictly speaking, because there was no reason to suppose the electron would possess spin. 'Has the electron spin?' was a question that simply had not been asked, and that could not be answered within the original model. But it was a question that at some point could easily enough suggest a fruitful line to follow.” (McMullin 1985, 263–4)

For McMullin, this accommodation was inspired by the theory itself and it harmoniously fitted with the program in its original formulation. The intrinsic creative suggestiveness of the program produced this adjustment. This ability to suggest modifications, McMullin argues, is a truth-indicative virtue of programs, i.e., it reveals that the content of the program is true or approximately true. That is, the ability of a program to survive to anomalies by means of changes that extend and improve its content reveals that the program gets something right about the real structure of the world.^7^

Daniel Nolan criticizes McMullin’s account of fruitfulness. Specifically, he claims that proven fertility can be reduced to other values and untested fertility’s importance is parasitic on the importance of other values. Indeed, a program has proven fertility if it is able to confirm itself through changes and this, Nolan argues, is a facet of the value of novel confirmation (1999, 271). On the other hand, he construes untested fertility as the chance that a future version of a program will perform better in fulfilling some virtues than its current version, such as strength and predictive power (ibid.). Still, despite being a dismissive, reductionist account, Nolan’s portrayal shares the core idea of fruitfulness as the power of programs to develop with Kuhn and McMullin’ accounts. Kuhn and McMullin construe fruitfulness as an ability of programs to develop by finding solutions to puzzles and anomalies. Nolan’s account, instead, focuses on the desirable development involved in novel confirmation and fulfilling certain virtues in the future.

More recently, McMullin’s analysis of fruitfulness has been criticized by Samuel Schindler (2017). In particular, Schindler argues that the discovery of the electron spin is not a genuine case of non-*ad hoc* accommodation (2017, 168). Although Schindler criticizes this historical case study, he claims that McMullin’s notion of fruitfulness should be developed further. In particular, the relation between fruitfulness and non-*ad hocness* should be studied since, Schindlers argues, non-*ad hocness* clearly is an important virtue of programs (2017, 170).

Nolan and Schindler provide the most recent accounts of fruitfulness. Although many recent studies have analysed the role of values in scientific practice (e.g. Douglas 2009; Steel 2010), they have largely ignored the literature on fruitfulness. In the next section, I develop the core idea of these accounts to provide an explication of fruitfulness and a strategy to assess programs.

### Developing the notion of fruitfulness

Let’s define fruitfulness as an *ability of research programs to develop*. A program develops when it enhances our knowledge or understanding of the world. In particular, fruitful programs develop by producing epistemic goods, such as novel hypotheses. Now, how can we assess this capacity to be productive? What are the relevant aspects for evaluating the productivity of programs? To answer these questions, I suggest focusing on the set of tools of research programs. This is what the researchers can use to extend and improve the content of a research program. Not any extension of the content counts as an improvement. For instance, making the content broader by formulating many implausible hypotheses does not make the program developing. The *quality* of the hypotheses generated should also be considered. Specifically, I suggest analysing two tools that scientists employ to extend programs’ content, i.e., *research questions* and *discovery heuristics*. These components are used to organize and plan research and to formulate novel hypotheses. They guide and constrain – together with other components – the development of research programs. Both the kinds of questions and heuristics and the ways in which they are employed are relevant factors to analyse for assessing the fruitfulness of programs.

Consider research questions first. Several studies have explored the importance of questions in the process of extending our knowledge (e.g. van Fraassen 1980; Mason 2002). For instance, some philosophers suggest understanding science as a question-answering process (Hintikka 1981). The questions we ask show what we find worthy of being investigated or what we think as a useful starting point for extending our knowledge. Examples of research questions are:

- What is the evolutionary function of jealousy for human beings?
- Is there a link between flu vaccines and miscarriage?

Now, why are research questions particularly relevant for assessing the *fruitfulness* of research programs?

Research questions constitute the starting point of the enquiry and, as Sven Lundstedt points out, “initial scientific questions, like first impressions, carry a great deal of weight in shaping the direction of a system of thought” (1968, 229). The research questions posed and the answers that they allow as appropriate significantly affect the development of research programs: questions can either promote or compromise the fruitfulness of programs.

Research questions have an important role in determining the methods used to conduct the research, which sizes of samples are legitimate, and what counts as relevant data (Mason 2002, 19).^8^ The choice of research questions depends on several factors, such as the background assumptions endorsed by the scientists or the scientific community. These assumptions can be of different sorts, e.g. moral, political, and methodological, and their importance can vary across contexts. For instance, question (b) may be the result of some moral assumptions, such as the idea that protecting human health is a moral duty and our research should then be focused on preventing damages caused by medicines. Such a moral concern may play little or no role in determining the question over the function of jealousy.

Analysing research questions can provide us with relevant information about the research conducted. Specifically, research questions have at least three dimensions that can be explored. First, examining a research question makes it possible to understand the *focus* of the research, i.e., the portion of the world that scientists want to investigate. Question (a) makes clear that human beings and their psychological traits are the phenomena on which the research primarily focuses. Second, research questions show the *aims* of the study. For instance, question (b) makes clear that the goal of such an investigation is gaining knowledge on the possible harms caused by some vaccines. Finally, by analysing research questions we can understand the *orientation* of the enquiry. Namely, research questions provide the direction of the investigation, i.e., the track along which the research can develop. Questions guide the choice of methodologies for formulating and testing hypotheses: the appropriateness of a method depends on the chances that such a method has of answering the question. Indeed, when it comes to answering a research question, some methods are appropriate, some others are not. To illustrate, let’s consider question (a). The aim of the study is investigating the function of jealousy and surveys and cross-cultural studies constitute – among others – appropriate methods for finding an answer. On the other hand, it is unlikely that using a cloud chamber helps to find an answer to this question.

In addition to constraining the choice of methods, research questions determine what a legitimate answer is. Here is where the relation between research questions and fruitfulness becomes clear. Not any sort of answer is good as a reply to a question. For instance, the answer “Jealousy is one of the causes of many stalking cases” to the question “What is the *function* of jealousy in evolution?” does not constitute a proper answer. That answer fails to address the specific aim of the question, namely clarifying the evolutionary history of jealousy. However, there is more to be discussed about the pertinence of answers. In a recent paper, Elisabeth Lloyd clarifies this point. She claims that “asking different questions makes contrasting classes of answers legitimate” and “each question carries with it an appropriate class of possible answers unique to it, and distinct from other contrasting classes of answers” (2015, 346). This means that research questions make some specific answers appropriate and some others irrelevant. As a consequence, while some answers are considered and analysed, others are ignored. This may negatively affect the development of research programs. Indeed, as Lloyd argues, a problematic use of research questions “can lead us to miss what’s really going on, therefore to scientific failure” (ibid.). By excluding some answers, questions can constrain research and have an impact on the effective development of research programs. I argue that some questions may perform better than others when it comes to  making a program fruitful. In the next section, a case study illustrates how the choice of research questions and the specific way in which they are used may either undermine or stimulate the development of programs.

Besides research questions, a good assessment of the fruitfulness of research programs should consider a further component of their set of tools, namely the discovery heuristics. These are the strategies used to formulate novel hypotheses. Heuristics guide, constrain, and help scientists’ imagination in the exploration of phenomena. They are not perfectly reliable strategies affording scientists good hypotheses in any context. However, they are taken as useful techniques to approach the world (Wimsatt 2007, 76). Assessing the discovery heuristics of a program, then, means evaluating its ability to produce epistemic goods.

What are the discovery heuristics that can make a program fruitful? Fruitful programs use *reliable* heuristics. That is, the pattern suggested by the heuristics for formulating hypotheses is rigorous, i.e., it does not involve defective reasoning. Reliable heuristics allow scientists to formulate *well-designed hypotheses*. This does not mean that reliable heuristics only produce hypotheses that turn out to be true. Rather, well-designed hypotheses are testable hypotheses that have good chances to be true. Moreover, it should be noted that heuristics producing *many* novel hypotheses do not necessarily make a program fruitful. Proliferation of hypotheses is not a mark of fruitfulness. Rather, it may indicate the sloppiness of programs.

The strategy for evaluating fruitfulness suggested in this paper makes it possible to distinguish between fruitful programs and “creative-but-stagnating” programs. It permits to draw a distinction between virtuous programs, whose content grows and improves over time, and vicious programs, whose content may grow by producing many hypotheses, but does not improve, i.e., programs that do not extend our understanding of the world. As mentioned, proliferation of hypotheses does not necessarily constitute desirable development.

To summarize, in this section I have presented the main accounts of fruitfulness and I have introduced my explication of fruitfulness and a strategy to assess programs. In the next pages, I provide an example unveiling the efficacy of this strategy.

## Fruitfulness at work: A case study

The adaptationist program is the leading research program in evolutionary psychology. It is led by the Santa Barbara School, which involves David Buss, John Tooby, Leda Cosmides, and many others. In order to distinguish such a program from other programs, I refer to this approach by using David Buller’s notation (2005) “Evolutionary Psychology” with capital letters.^9^ Evolutionary Psychology is committed to *methodological* adaptationism. In a seminal paper, Paul Godfrey-Smith distinguishes various kinds of adaptationism and defines methodological adaptationism as the thesis that

“The best way for scientists to approach biological systems is to look for features of adaptation and good design. Adaptation is a good “organizing concept” for evolutionary research” (Godfrey-Smith 2001, 337).

Looking for adaptations is the most efficient strategy to extend our body of knowledge. Adaptations are the products of natural selection: traits that solved specific adaptive problems faced by our ancestors in the Environment of Evolutionary Adaptedness (EEA). Methodological adaptationism does not necessarily involve the assumption that adaptation is the most widespread product of evolution. In other words, adaptationist programs are not necessarily committed to *empirical* adaptationism, i.e., the idea that natural selection has caused most – if not all – of the psychological traits. In this view, adaptations are the primary, omnipresent component of the biological world (Godfrey-Smith 2001, 336). Methodological adaptationism, instead, does not tell anything about the actual composition of the biological world. Its focus is the best way to explore it. Starting out the research by focusing on adaptations is, according to methodological adaptationists, the most effective and reliable approach to understand the evolutionary origin of traits, regardless of the actual amount of adaptations.^10^ As Cosmides and Tooby explain, methodological adaptationism is essential to Evolutionary Psychology: “if one is interested in uncovering intelligible organization in our species-typical psychological architecture, discovering and describing its adaptations is the place to begin” (1992, 55).

Adaptationist psychologists have proposed plenty of hypotheses to explain a wide range of traits. They often stress the productivity of their approach. Indeed, the praise of adaptationism’s ability to account for previously ignored phenomena and make discoveries is a *leitmotif* found in many papers (e.g. Lewis et al. 2017; Buss and Schmitt 2011). For instance, Stephen Pinker (2002) claims that “in the study of humans, there are major spheres of human experience – beauty, motherhood, kinship, morality, cooperation, sexuality, violence – in which evolutionary psychology provides the only coherent theory” (2002, 135). Buss and Reeve mention this exceptional virtue of their approach:

“By any reasonable standard, evolutionary psychology has discovered an impressive array of empirically documented phenomena that were not discovered by prior mainstream nonevolutionary psychologists” (Buss and Reeve 2003, 849).

To prove that, Buss and Reeve provide a long list of phenomena explained by Evolutionary Psychology, such as desire for sexual variety, stepchild abuse, and causes of divorce (ibid., 850).

Prima facie, Evolutionary Psychology seems to be an extremely fruitful research program. It is able to develop its content by suggesting many hypotheses that improve our understanding of the evolutionary origin of psychological traits. Evolutionary Psychology is portrayed as a constant blooming of new hypotheses and methodological adaptationism looks like an exceptionally fruitful strategy. Psychologists claim that their approach is so fruitful because it constitutes the most reliable and non-arbitrary way to classify and understand phenomena (Buss 1995, 6). Moreover, they argue that alternative approaches to the adaptationist program do not equal such a great triumph in extending our knowledge. In their view, this program has clearly shown its complete dominance in developing evolutionary psychology (Buss and Kern Reeve 2003, 852).

Evolutionary Psychology has also been criticized because of its astonishing productivity. Many scholars challenge the overuse and soundness of adaptationist explanations (Richardson 2007). In their view, such a large amount of hypotheses would be a sign of the unreliability and sloppiness of methodological adaptationism. So, is Evolutionary Psychology fruitful? How can we deal with this criticism? Let’s assess Evolutionary Psychology’s fruitfulness by analysing two components of its set of tools, namely its research questions and discovery heuristics.

### Research questions in Evolutionary Psychology

What are the research questions guiding Evolutionary Psychology? Buss provides us with an answer. The questions are:

“What is the nature of the psychological mechanisms that evolution by selection has fashioned? Why do these mechanisms exist in the form that they do – what adaptive problems did they arise to solve, or what are their functions?” (Buss 1995, 5).

The reference to natural selection and function makes it clear that these are *adaptationist* questions, which can be exemplified by the following question:

(AQ) What is the function of trait T?

Asking about the *function* of a trait means enquiring its evolutionary function. The question, then, revolves around adaptations. Following the aforementioned three dimensions of research questions, it is possible to clarify some aspects of this question. The *focus* of the adaptationist question are biological traits. Its *aim*, then, is gaining knowledge on the evolutionary origin of these traits. Specifically, the question addresses the role of natural selection in the development of traits. Finally, the *orientation* of this question is adaptationist, i.e., the starting point is assuming that the trait studied is an adaptation. Psychologists see asking this question as the most helpful procedure to advance our understanding of the role of evolution on psychological mechanisms. If the adaptationist question was not being used, many discoveries would not be possible (Cosmides and Tooby 1994, 43).

Recently, Elisabeth Lloyd has criticized the use of adaptationist questions. She argues that such questions impoverish the quality of research in evolutionary biology and psychology. In her view, the adaptationist questions as used in Evolutionary Psychology only admit adaptive answers, namely answers involving functions and adaptations (Table 1).

**Table 1:** Adaptationist question and its answers (Lloyd 2015, 346)

Question	Answers
What is the function of the trait T?	A: The function of this trait is F. A: The function of this trait is G. Etc.

This means that this strategy rules out other possible answers, i.e., answers involving nonadaptive forces. Indeed, Lloyd claims that Evolutionary Psychologists see adaptive and nonadaptive answers as mutually exclusive. In particular, they see nonadaptive hypotheses as statistical nulls. They understand “the rejection of adaptive hypotheses as logically necessitated for consideration or acceptance of any nonadaptive hypothesis” (2015, 349). This is evident in the discussion of Evolutionary Psychology’s methodology offered by Buss and colleagues (Buss et al. 1998). They claim that when an adaptationist hypothesis is not supported by data, the following options are available to psychologists. First of all, researchers may want to check whether the tests have been done properly. Second, if the tests have been correctly done and the adaptationist hypothesis is just wrong, researchers can suggest alternative adaptationist hypotheses. Third, researchers may want to test non-adaptationist hypotheses. However, considering non-adaptationist hypotheses, Buss and colleagues argue, involves trying “to disconfirm all existing functional explanations” (Buss et al. 1998, 544). This – Lloyd argues – is misunderstanding how evolution works. Several evolutionary factors can be involved in the evolution of a trait, i.e., several answers can *simultaneously* account for a trait. The way Evolutionary Psychologists use the adaptationist question leads to ignore important evolutionary factors, such as byproducts, that need to be considered to provide an exhaustive analysis of phenomena. As a consequence, the power of this program to provide adequate explanations is undermined. Lloyd states that the fruitfulness of this inquiry improves with a different question: “What evolutionary factors may play a role in the form and structure of the trait T?” (2015, 346) (Table 2).

**Table 2:** Evolutionary factors question and its answers (Lloyd 2015, 346)

Question	Answers
What evolutionary factors may play a role in the form and structure of the trait T?	A: This trait occurs in the population because it has the function F, i.e., the trait is an adaptation. A: This trait occurs in the population because it has the function G, i.e., the trait is an adaptation. A: This trait occurs widely in this population because it is genetically linked to a trait that is highly adaptive in this species (genetic linkage or correlation). A: This trait has its current form largely because of an ancestral pattern (phyletic inertia). A: This trait has its current form and distribution because of pleiotropy with a trait that was under natural selection (pleiotropy or byproduct). A: This trait has its current form and distribution because it is a byproduct or bonus of a trait that is strongly selected in the opposite sex in this species (byproduct or bonus of an adaptation). A: This trait has its current form and distribution because of some combination of the above factors. Etc.

Nonadaptive and adaptive answers here are not seen as mutually exclusive. Namely, the question does not demand a rejection of adaptive answers in order to make room for nonadaptive ones. While the adaptationist question underestimates the actual role of different factors in evolution, the evolutionary factors question allows for different kinds of explanation and it is able then to better account for the complexity of evolution. The two questions differ in their orientations. The adaptationist question has an adaptationist orientation and sees assuming that a trait is an adaptation as the best strategy to study it. The evolutionary factors question, instead, does not make such an assumption and it involves a pluralistic methodology. This question makes it possible to offer a pool of stronger hypotheses that may enhance the fruitfulness of evolutionary psychology.

If Lloyd is right, the adaptationist question, as used in Evolutionary Psychology, may impede the desirable development of the program. In other words, the possibility of extending and improving the content is undermined by the use of a limiting question that ignores legitimate answers, namely answers that, because of the complexity of evolution, need to be considered. This means that by *monopolizing* the kind of answers pursued, the adaptationist question does not make Evolutionary Psychology a fruitful program. The use of this question may lead to what Philip Kitcher calls the “tyranny of the ignorant” (2003, 130). Kitcher argues that because of some moral, political background assumptions, some questions are often undervalued by scientists. The case study shows that important questions, such as the pluralistic one suggested by Lloyd, are ignored and this negatively affects the advancement of science. The tyranny of the adaptationist question in Evolutionary Psychology is not due to moral and political assumptions. Rather, it is attributable to the extreme confidence in the adaptationist approach as a reliable strategy to improve our understanding of the evolution of human psychology. That is, in this case the tyranny of the ignorant is due to methodological assumptions.

### Heuristics in Evolutionary Psychology

The second factor I suggest for analysing the fruitfulness of research programs are the discovery heuristics, namely the strategies for developing novel hypotheses.

Evolutionary Psychologists apply two main heuristics to suggest hypotheses, i.e., reverse engineering and adaptive thinking (Griffiths 1996, 514). Reverse engineering “involves working backwards from what we know of human psychological architecture, to what its evolutionary functions might have been in ancestral environments” (Durrant and Haig 2001, 362). Scientists detect a human beings’ trait and imagine its selective advantage for our ancestors. For instance, if they have data about marital jealousy, psychologists formulate a hypothesis that explains such a trait as a solution to an adaptive problem faced by our ancestors in EEA. When using adaptive thinking, instead, researchers conceive “the adaptive problems ancestral humans were likely to have faced and generate plausible accounts for the psychological mechanisms which would have evolved to solve them” (ibid.). That is, scientists imagine a particular adaptive problem in EEA, such as protecting one’s own offspring, and they formulate a hypothesis concerning the existence of a certain adaptation solving that problem, such as jealousy. The products of these heuristics are adaptationist hypotheses, i.e., hypotheses that explain traits as adaptations. Indeed, both reverse engineering and adaptive thinking are adaptationist strategies that ascribe to natural selection the primary role in the explanation of traits.

Evolutionary Psychologists have firmly defended the adequacy of these heuristics. They argue that, despite being a young field, Evolutionary Psychology has proven itself capable of suggesting plenty of evolutionary hypotheses and advancing our understanding of human psychology and that the adaptationist methodology has a crucial role in making this possible. For instance, Cosmides and Tooby claim that the adaptationist methodology “can free cognitive scientists from the blinders of intuition and folk psychology, allowing them to construct experiments capable of detecting complex mechanisms they otherwise would not have thought to test for” (1994, 41). Focusing on the evolutionary function of traits is seen as “an indispensable methodological tool, crucial to the future development of cognitive psychology” (ibid., 43). According to several Evolutionary Psychologists, this is exactly the purpose of Evolutionary Psychology. As Schmitt and Pilcher (2004) explain, Psychologists take the view that “identifying all the psychological adaptations that make up human nature – the adaptationist program of humanity – is what evolutionary psychology should be all about” (2004, 644). Recently, a group of Evolutionary Psychologists has published a guide for conducting research in Evolutionary Psychology (Lewis et al. 2017). In this guide, Psychologists confirm their confidence in adaptive thinking and reverse engineering. Indeed, these heuristics are suggested as the appropriate, reliable methodological tools for generating rigorous hypotheses (355).

Do these discovery heuristics make Evolutionary Psychology fruitful? As mentioned, fruitful programs use *reliable* heuristics, namely heuristics that avoid defective reasoning. Let’s analyse the reasoning involved in reverse engineering and adaptive thinking.

Reverse engineering involves observing a current trait and inferring from it the adaptive problem that the trait possibly solved in EEA. Adaptive thinking involves first hypothesizing an adaptive problem faced in EEA, and then inferring from that the specific trait that was evolved to solve the problem. Both these heuristics assume “a strong relationship between adaptive forces and the resulting organism” (Sterelny and Griffiths 1999, 242). This assumption of optimality may hinder the formulation of well-designed hypotheses. Formulating well-designed hypotheses on the evolutionary nature of psychological traits is not an elementary work. The evolution of traits involves several aspects that should be meticulously considered in the formulation of hypotheses. For instance, developmental and environmental factors do play a role in the evolution of traits by constraining the action of natural selection. Because of such a strong optimality assumption, it is not clear how these heuristics can account for these factors. As Paul Griffiths (2001) explains, the inferences allowed by these heuristics (i.e. inferring the adaptive problem from the solution and inferring the solution from the adaptive problem) are then unreliable. These heuristics may be too imprecise to constitute reliable tools for formulating evolutionary hypotheses. This means that they may constitute an obstacle to the development of the research program. Lewis and colleagues have recently argued that Psychologists do consider various evolutionary factors in the formulation of hypotheses, such as byproducts (Lewis et al. 2017, 362). However, whether the consideration of these factors is widespread as claimed should be assessed by means of a careful analysis of the hypotheses suggested by Psychologists in their papers. In order to be accurate, the consideration of different evolutionary factors should be present in *any* evolutionary study of psychological traits.

Recently, Edouard Machery (2011) argued that although it might be true that the adaptationist heuristics may produce inaccurate hypotheses, this does not mean that they systematically do that. He claims that the adaptationist strategy may actually be beneficial, since it reduces the class of possible hypotheses. Why is this beneficial? Machery does not clarify the benefits of reducing the class of hypotheses. However, there are at least two reasons why this may be beneficial. First, it makes scientists’ work easier. Having a restricted number of hypotheses to analyse makes it easier to find out what is the best hypothesis among them. Second, this strategy may be beneficial because it admits only the hypotheses that are worth considering. It rules out bad hypotheses, namely hypotheses that are likely to be false. One of the benefits of this strategy is then preventing proliferation of hypotheses.

In fact, these heuristics prevent the formulation of many hypotheses. Is this a virtue of the program? As mentioned above, these heuristics face the problem of focusing exclusively on adaptationist hypotheses and ignoring other kinds of evolutionary hypotheses, such as the ones involving byproducts. This means that they ignore plausible evolutionary hypotheses that need to be considered to reach an exhaustive analysis of the evolution of traits. This is not a desirable reduction of hypotheses. Moreover, these heuristics may actually multiply hypotheses by suggesting too many adaptationist hypotheses, also the ones that are likely to be false but are considered because of their adaptationist nature. The adaptationist strategies, then, do not prevent the proliferation of hypotheses. They actually *encourage* it. By preventing the formulation of many legitimate evolutionary hypotheses, these strategies undermine the possibility to improve and extend our understanding of human psychology and this is a problem for the fruitfulness of Evolutionary Psychology.

The discovery heuristics used in Evolutionary Psychology have what Gould calls “virtuosity in invention” (1978, 530). They are powerful tools enabling scientists to imagine scenarios explaining the existence of a trait and its evolutionary function. Still, because of the unreliable inferences they allow, their ability to formulate well-designed hypotheses is undermined. Their power to develop Evolutionary Psychology is then limited.

### Is Evolutionary Psychology fruitful?

The previous sections pinpoint the problems of the research question and heuristics used in Evolutionary Psychology. These problems constrain the power of the program to extend and improve the understanding of human psychology and, then, they compromise its fruitfulness. Still, there is room for improvement.

The adaptationist question is not necessarily fruitless. For instance, in evolutionary medicine this question is used as a fruitful tool to improve our understanding of medical conditions and, in some cases, our ability to cure diseases (Nesse and Stearns 2008). The adaptationist question is just one of the evolutionary questions used to guide research in evolutionary medicine. Indeed, as Randolph Nesse and Stephen Strearns explain, research in evolutionary medicine involves both the adaptationist question “How has mechanism M given a selective advantage to individuals?” and the general evolutionary question “What is the phylogeny of this mechanism?” (Nesse and Stearns 2008, 30). Evolutionary Psychology, then, might improve its fruitfulness by employing a more pluralistic methodology, namely involving both adaptationist and non-adaptationist questions. The evolutionary factors question suggested by Lloyd seems to be a good option to improve Evolutionary Psychology’s fruitfulness. Moreover, adaptationist heuristics are not doomed to failure in any context. By analysing research in evolutionary biology, Sara Green noticed that while methodological adaptationism may be a productive heuristic for some specific tasks, such as exploring the connections between causal capacities and selective pressures, it performs poorly for other tasks, such as studying drift (2014, 493). The same might be true for Evolutionary Psychology. It is then necessary to understand in which cases adaptationist heuristics may be able to develop the program and in which cases they hinder the development.

Psychologists often praise the fruitfulness of their program. They frequently provide lists of the successes of Evolutionary Psychology, such as new discoveries and theories (e.g. Buss and Reeve 2003). It is an open question whether all the items on these lists do constitute desirable development, i.e., extensions of our understanding of human psychology. Indeed, many of the discoveries and theories of the program have been criticized as being the products of poor scientific research, even the ones that are usually presented as triumphs of the program, such as the Sexual Strategies Theory suggested by Buss and Schmitt (1993). Evolutionary Psychology is a very creative program. Still, its research question and heuristics compromise its fruitfulness.

## Conclusion

I have argued that the traditional accounts of fruitfulness are too vague to provide a strategy to assess the fruitfulness of research programs. I have suggested a new approach, which is focused on analysing the set of tools used by scientists to extend and improve the content of a program. Specifically, this approach focuses on examining research questions and discovery heuristics.

What does the analysis of questions and heuristics tell us about research programs? What does fruitfulness reveal about research programs? To answer this question, it is useful to recall the traditional distinction between epistemic and cognitive values (e.g. Laudan 2004). While epistemic values are truth-indicative features of programs and inform us about their truth value, cognitive values do not tell anything about the truth value of programs, but are valuable for pragmatic reasons, such as making research easier and faster. If this distinction holds, what kind of value is fruitfulness? Some philosophers argue that it is an epistemic value (McMullin 1982), while others claim it is a cognitive value (Kuhn 1977; Douglas 2009). I argue that fruitfulness is a cognitive value. More precisely, fruitfulness is one of the values that Daniel Steel calls extrinsic epistemic values, i.e., values promoting “the attainment of truth without themselves being indicators or requirements of truth” (Steel 2010, 18). Fruitfulness is a relevant value because it is an essential virtue of *scientific* research programs, which have to be able to develop and improve our understanding or knowledge of the world. Moreover, fruitfulness may facilitate the fulfilment of other values. Using research questions that permit an exhaustive analysis of phenomena and heuristics that enable scientists to formulate well-designed hypotheses promotes the fulfilment of values like predictive power, scope, and explanatory power. Nonetheless, the fact that programs use reliable methods does not inform us about their truth value.

Despite having an explication of fruitfulness and a strategy to assess it, scientists’ assessment of programs may still clash with each other. For instance, scientists may disagree on which research questions are more reliable. Nonetheless, having precise criteria – such as questions and heuristics – provides the basis for unambiguous and useful debates. As noticed in the second section, the accounts of fruitfulness suggested in the literature are too vague to do this. The strategy to assess fruitfulness suggested in this paper makes it possible to avoid several disputes over the merits of programs: to be fruitful, programs have to use suitably detailed research questions and reliable discovery heuristics.

Various factors may contribute to the fruitfulness of programs, such as local contingencies and the skills of scientists. For instance, the funds available to programs and scientists’ competence can contribute to determine the development of programs. Hence, considering these aspects may be important for an accurate assessment of the fruitfulness of programs.

In this paper, I explicated fruitfulness as the power of programs to develop. Moreover, I suggested a strategy to assess the fruitfulness of programs. This analysis may be developed further by examining other tools that can be used to improve and extend the content of programs, such as the methodologies to test hypotheses and predictions. However, the explication and strategy I suggest constitute a starting point for a serious discussion of the actual relevance of fruitfulness in the assessment of research programs.

## Footnotes

1 Longino also suggests replacing fruitfulness with two alternative values, namely applicability to current human needs and diffusion of power (Longino 1995, 389).

2 Talking about “research programs” recalls Imre Lakatos’ philosophy. However, I am not committed to the specific aspects of the Lakatosian notion of research program, such as hard-core and protective belt. I construe a research program as a set of theories that share some fundamental assumptions and methodologies.

3 This problem affects many values. As mentioned in the introduction, several papers discuss how to best define simplicity (e.g. Forster and Sober 1994; Baker 2003). It is beyond the scope of this paper to address the problems arising from the vague definitions of other values.

4 While Kuhn mentions theories and paradigms in his analysis of fruitfulness, I talk about programs. Although these are not synonyms, their differences do not constitute a problem for my analysis of fruitfulness.

5 McMullin employs the term “fertility” to refer to the virtue discussed in this paper. In the literature, philosophers also use “fecundity” (Quine 1955) and “boldness” (Colyvan 1999).

6 The focus on proven fertility was dictated by McMullin’s purpose of employing fruitfulness to defend scientific realism (1979).

7 Recently, Schindler (2017) has pointed out that the non-*ad hoc* modifications analysed by McMullin are akin to Imre Lakatos’ positive heuristic (Lakatos 1970). They both involve adjustments that do not seriously alter what Lakatos calls the “hard core” of the program, but they are able to extend its content. Schindler calls these modifications “de-idealisations of the theory” (2017, 156).

8 See Bryman (2007) for a critique of the idea of research questions as fundamental factors for research design.

9 Machery and Clark Barrett (2006) argue that any distinction made between the researches conducted in evolutionary psychology is problematic because they often share methodologies and topics. It is beyond the scope of this paper to discuss this issue. However, the research discussed in this paper is the one committed to methodological adaptationism.

10 Methodological adaptationism comes both in a strong and a weak form. While strong methodological adaptationism sees the adaptationist strategy as the only successful approach for understanding evolution, weak methodological adaptationism pictures it as just one of the successful strategies (see Lewens 2009, 169).
